# Control Work

**Published:** 1895-12

**Authors:** 


### CONTROL WORK.

*California.* The Board of Health of San Francisco has taken determined steps looking to a better milk-supply for her people. The movement has the support of the *Pacific Medical Journal*, an influential publication on the Western coast. They demand the destruction of every tuberculous cow. Other towns on the Pacific coast will follow in the lead. The following rules have been adopted by the Board of Health of San Francisco.

I. The Board of Health places the standard at a specific gravity of not less than 1.029; total milk-solids at not less than 12 per cent., and of butter not less than 3 per cent.

II. Milk containing more than 88 per cent. of water or less than 12 per cent. of milk-solids, of which not less than 25 per cent. must be butter-fat, shall be declared adulterated, impure, and unwholesome.

III. The following table of relative percentages of the respective months is hereby adopted by the Board of Health: January and February, 9½ per cent.; March, 9 per cent.; April, May, and June, 9½ per cent.; July, August, and September 10, per cent.; balance of the year, 10½ per cent.

It shall be the duty of the milk-inspector to inspect all places where milk is stored or kept for sale, all wagons, carriages, or other vehicles, railroad cars, or conveyances of any other kind used for the conveyance or transportation or delivery of milk to any warehouse, dairy, hotel, factory building, farm, stable, railroad depot, steamboat landing, establishment of any kind; to inspect all vessels, cans, packages, refrigerators, or receptacles of milk, and to take samples therefrom not exceeding one quart for the purpose of inspecting, testing, and analyzing the same.

It shall be the duty of the milk-inspector to obtain two samples of milk to be analyzed, both of which shall be sealed, one deposited at the health office and one left with the party from whom the milk was obtained. Milk which does not come up to the standard herein adopted by the Board of Health of the city and county of San Francisco shall be condemned, seized, and destroyed by the milk-inspector.

It shall be the duty of the milk-inspector to arrest and prosecute any and all persons engaged in the sale, exchange, or distribution, or who shall sell, exchange, or distribute any adulterated, impure, or unwholesome milk, as provided for in the ordinance, Milk-tests adopted by the Board of Health: "The specific gravity shall be determined by any properly constructed lactometer, and percentage of cream shall be determined by any properly constructed creamometer or lactoscope, and the butter-fat shall be measured by the so-called Babcock test. The total solids shall be computed by the formula of Hehne and Richmond. The color-tests shall be uniform to the tests established by the pioscope of Heeren."

San Francisco finds among her new industries two establishments for the preparation of equine flesh for consumption as a food-product among her people. Great care should be exercised by health authorities in not asking of these places any different rules than those which apply to other abattoirs and slaughter-houses beyond the fact of so marking the meat as to the species from which it is obtained and that it is from subjects not diseased in any way prejudicial to health. For it must be remembered that no better or cleaner flesh exists than that of a horse, and mere prejudice must not decide the action of such bodies.

*Indiana.* The departments of health and police have been investigating the horse-slaughtering establishment at Hammond, and claim to have found some evidence of diseased meat being prepared for market. They have called into service three expert veterinarians, and a rigid examination will be made.

*Iowa.* The work of the State Veterinarian has been suspended, owing to the exhaustion of the funds available for this work. The appropriation for 1896 will be available on January 1st, when the work will be resumed.

Hog-cholera is unusually prevalent in Iowa, and causing very serious losses to her people. Prominent breeders and others are beginning to look toward the promised legislation for relief. Measures looking toward its better control and restriction will be asked for.

The State Legislature will be asked at its coming session to create laws for the better protection of her veterinary graduates. We sincerely trust that it will be in the direction of a State board of examiners, as all other measures for registration are bound to defeat the objects desired, and are difficult and expensive to enforce. A form of license should be created and registration under this plan through said board obtained.

*Massachusetts.* The New England Cattle Commission held an important meeting at Providence, R. I., on November 21 and 22, 1895. The following subjects were considered: "Tuberculin," by C. M. Winslow, Brandon, Vt.; "Disinfection," by Victor I. Spear, Braintree, Vt.; "The Relation between Bovine and Human Tuberculosis," by F. A. Rich, M.D., V.S., Burlington Vt.; "Legislation," by F. H. Osgood, Boston, Mass.

*New York.* A very important meeting was held at the New York Academy of Medicine on the evening of November 8th, for the consideration of "The Relation of Bovine to Human Tuberculosis." The several divisions of the subject were assigned to the following well-known veterinarians and physicians: "Tuberculosis as Viewed by a Veterinarian,"[1] Prof. H. D. Gill, of the New York College of Veterinary Surgeons; "The Work of Foreign Veterinarians in Tuberculosis,"[2] Prof. Leonard Pearson, of the University of Pennsylvania; "The Control of Tuberculosis in Massachusetts,"[3] Prof. F. H. Osgood, of Harvard University; "The Best Methods for the Eradication of Tuberculosis,"[4] Prof. James Law, of Cornell University; "The Transmission of Tubercle Bacilli and Tubercular Products in Milk," Prof. Harold C. Ernst, of Harvard Medical School; general discussion by Drs. George B. Fowler, William H. Thompson, A. Jacoby, Edward W. Martin, Ernest J. Lederle, and others.

*Pennsylvania.* A very serious outbreak of hog-cholera prevails in the vicinity of Pottstown, and only local measures are

[1] Page 804. [2] Page 797. [3] Page 791. [4] Page 808.

in force for its restriction. The Live Stock Sanitary Board of Pennsylvania, which should have been organized last June, in accordance with the provisions of the act creating this body, still remains incomplete, owing to the failure of the Governor to appoint a State Veterinarian. The probable outcome of this delay will be numerous appeals to the Legislature for reimbursement for losses or law-suits against the State for damages sustained.

*Oregon.* The horse-meat slaughtering establishment just outside of Portland has commenced work on a large consignment of horses. It is said that only hams will be prepared at this establishment for foreign shipment, and other animal extracts and products will be produced from the remainder of the carcass.

*Wisconsin.* State Veterinarian Scott reports very serious losses among the horses of Northern Wisconsin from eating golden-rod, and quotes this as an objection why it should not be made our national flower.

